# Spatial Transcriptome Analysis of B7-H4 in Head and Neck Squamous Cell Carcinoma: A Novel Therapeutic Target for Anti-Immune Checkpoint Inhibitors

**DOI:** 10.1007/s12105-025-01815-w

**Published:** 2025-06-30

**Authors:** Yuri Noda, Masao Yagi, Koji Tsuta

**Affiliations:** 1 Department of Pathology and Laboratory Medicine, Kansai Medical University Hospital, 2- 3-1 Shin-machi, Hirakata, Osaka 573-1191 Japan; 2 Department of Pathology, Kansai Medical University, 2-5-1 Shin-machi, Hirakata, Osaka 573-1010 Japan; 3 Department of Otolaryngology, Head and Neck Surgery, Kansai Medical University Hospital, 2-3-1 Shinmachi, Hirakata, Osaka 5731191 Japan

**Keywords:** Antibody–drug conjugate, B7-H4, Immune checkpoint inhibitor, PD-L1, Squamous cell carcinoma

## Abstract

**Purpose:**

A significant proportion of patients with head and neck squamous cell carcinoma (HNSCC) are ineligible for immune checkpoint inhibitors (ICIs) because of low programmed cell death protein-ligand 1 (PD-L1) expression. The therapeutic potential of B7-H4 (*VTCN1*) was investigated using immunohistochemistry (IHC) and spatial transcriptomics (ST).

**Methods:**

IHC analysis of B7-H4, PD-L1, CD3, CD4, and CD8 was performed using a tissue microarray [94 HNSCC, 94 adjacent squamous intraepithelial neoplasia (SIN), and 69 adjacent normal oral mucosa (NOM) samples]. B7-H4 and PD-L1 expression levels were assessed using tumor cell score (TC; positive, TC > 1%), immune cell score, and combined positive score. ST was performed on six HNSCCs with paired SINs and NOMs to confirm the expression and distribution of B7-H4 (*CTVN1*), PD-L1 (*CD274*), CD4 (*DC4A*), and CD8 (*CD8*).

**Results:**

In HNSCCs, TCs revealed a mutually exclusive B7-H4/PD-L1 expression pattern in 55% of samples (*p* < 0.05). B7-H4 positive TCs were more frequent in HNSCCs (79%) than in SINs (10%) and NOMs (2%). ST analysis confirmed mutually exclusive *VTCN1*/*CD274* upregulation in 83% of samples (*n* = 6) and demonstrated co-localization of B7-H4 protein and *VTCN1* in IHC-positive areas. B7-H4 was significantly correlated with low-CD8^+^ T-cell infiltration (*n* = 94, *p* = 0.009), and *CD8A* mRNA was down-regulated in the *VTCN1*^+^ area compared with that in the *VTCN1*^+^ area.

**Conclusion:**

B7-H4 is a promising antibody–drug conjugate target in ICI-resistant HNSCC. IHC combined with TCs enabled the reliable assessment of B7-H4, given its co-localization with *VTCN1* in IHC-positive areas and association with low-CD8^+^ T-cells.

**Supplementary Information:**

The online version contains supplementary material available at 10.1007/s12105-025-01815-w.

## Introduction

Head and neck squamous cell carcinoma (HNSCC) is the seventh most common cancer worldwide, and by 2040, new cases are projected to increase by nearly 50% [1, 2]. Despite the increase in incidence, the 5-year survival rate has remained at approximately 60% [3] due in part to the complexity of the available treatment options [4]. Immune checkpoint inhibitors (ICIs), such as programmed cell death protein-ligand 1 (PD-L1)/programmed cell death protein 1 (PD-1), can be used in therapies to prolong the outcome of progressive and platinum-refractory HNSCCs [5–10]; however, the objective response rate is low, at approximately 15% of patients with HNSCC [11]. This is because approximately 80% of patients with HNSCCs are classified as ineligible due to low PD-L1 expression [5–10], and approximately 24% of HNSCC cases may include “immune-cold” tumors exhibiting low immune cell infiltration and immune activity [11]. To overcome these limitations, current research is focused on developing novel evaluation systems for eligible patient selection and strategies that combine ICIs with chemoradiation or investigate the potential of novel antibody–drug conjugates (ADCs). Therefore, novel targets and new evaluation methods are required for HNSCCs with ICI resistance and low PD-L1 expression.

In this context, B7 homolog 4 (B7-H4, *VTCN1*) has emerged as a promising therapeutic target. As a member of the B7 family, which includes PD-L1 [12, 13], B7-H4 is implicated in the epithelial–mesenchymal transition via the Wnt–β-catenin pathway in tumors, and it may inhibit CD4^+^ and CD8 + T-cell proliferation and cytokine production through the ligation of an unknown receptor expressed by activated T cells [12, 14]. Notably, B7-H4 is overexpressed in tumor cells under immune-desert conditions [14–16] and shows mutually exclusive expression against PD-L1 in various cancers [15, 17–22]. Given these characteristics, along with its low or absent protein expression in normal tissues, B7-H4 may represent a potential target for enhancing the efficacy of anti-PD-1/PD-L1 therapies [12, 13]. Several ADCs targeting B7-H4 are currently being developed, and clinical trials of B7-H4-blocking antibodies are underway [23, 24]. However, the single-cell level protein and corresponding mRNA expression, distribution of B7-H4, and its association with tumor-infiltrating T cells in HNSCC have not yet been elucidated.

To assess the potential of B7-H4 as a novel therapeutic target in HNSCC, it is essential to establish highly sensitive evaluation methods that correspond with mRNA (*VTCN1*) expression and distribution. Recent research identified the 73 − 10 clone as having superior sensitivity and specificity for detecting PD-L1 among five companion or complementary clones [25–27]. Visium spatial transcriptomics also offers the advantage of observing gene expression within tissue sections while preserving tissue architecture, thereby linking mRNA expression to its original location [28].

This study investigated the potential of B7-H4 as an effective ADC in patients with HNSCC for whom PD-L1 is not a valid therapeutic target. The expression levels and distribution of B7-H4 (*VTCN1*), PD-L1 (*CD274*), CD4 (*CD4*), and CD8 (C*D8A*) were examined using immunohistochemistry (IHC) and spatial transcriptomics (ST) analysis of the HNSCCs, adjacent normal mucosa (NOM), and squamous intraepithelial neoplasia (SIN). Clinicopathological analysis was performed using the B7-H4 IHC results to examine the association between T-cell infiltration and other microenvironmental factors.

## Materials and Methods

### Patients

The patient cohort for this study was derived from our previously published work [29]. This retrospective study included 100 patients. Of these, 94 patents were clinicopathologically analyzed, and 6 were enrolled in the Visium analysis. All patients were diagnosed with progressive HNSCCs and underwent surgical resection without prior chemotherapy or radiation therapy at the Department of Otorhinolaryngology, Head and Neck Surgery, Kansai Medical University Hospital, between January 2009 and December 2024. This study was approved by the Institutional Review Board (approval number: 2023024). Additional demographic and clinicopathological characteristics are provided in Online Resource 1 and 2.

### Construction of Tissue Microarrays (TMAs) for Clinicopathological Analysis Using IHC

In total, 1,040 cores were collected to construct a TMA for clinicopathological analysis using IHC. This included four cores from each of the 94 HNSCC samples, 94 adjacent SIN samples, and 62 adjacent NOM samples from FFPE-resected HNSCC tissues from 94 patients. Four HNSCC cores were collected from different invasive areas: two from superficial invasive areas (depth of invasion ≤ 5 mm) and two from areas of deeper invasion (depth of invasion > 6 mm). Each FFPE tissue block was sampled with 2.0-mm cores using a tissue-arraying instrument (Azumaya Corporation, Tokyo, Japan). We excluded carcinoma in situ (CIS) from the analysis, and all cores had ≥ 100 epithelial cells. Our previous study [29] provides a detailed summary of the results.

### Histopathological and Clinicopathological Analysis for IHC Using TMAs

The clinicopathological features of the patients were described in our previous study [29] and in Online Resource 1. IHC analyses of the TMA tissue sections were performed using antibodies against B7-H4 (D1M8I, 1:200; Cell Signaling Technology, Inc., MA, USA), 73 − 10 (pre-diluted; Leica Biosystems, Newcastle Upon Tyne, UK), CD3 (PS1, pre-diluted; Nichirei Bioscience, Inc., Tokyo, Japan), CD4 (1F6, pre-diluted; Nichirei Bioscience, Inc.), and CD8 (G2B10, 1:20000; Proteintech Group, Inc. IL, USA). For B7-H4, antigen retrieval was performed using ethylenediaminetetraacetic acid or citrate buffer at 95 °C for 1 h, followed by detection using a Histofine Simple Stain MAX-PO^®^ polymer detection system (#NIC-414131 F; Nichirei Bioscience Inc.) and visualization using diaminobenzidine. The 73 − 10, CD3, CD4, and CD8 were visualized as previously described [29].

The expression of B7-H4 and 73 − 10 was evaluated based on the tumor cell score [TC, also known as tumor proportion score (TPS)], immune cell score (IC), and combined positive score (CPS) [30–32]. Traditionally, these evaluation methods used the invasive carcinoma components as the numerator and denominator. However, this study used the non-neoplastic epithelium for NOM and the dysplastic components for SIN while maintaining the original method for invasive carcinoma components in HNSCC. TC, IC, and CPS values ≥ 1% were considered positive, and values ≤ 0% were considered negative. The mutually exclusive expression of B7-H4 and 73 − 10 was noted as follows: B7-H4^*+*^ and 73 − 10^*−−*^ or B7-H4^*−−*^ and 73 − 10^*+*^. The percentage of CD4^+^ and CD8^+^ tumor-infiltrating lymphocytes (TILs) was measured and classified as low (< 20%) or high (≥ 20%) based on CD3^+^ areas, and cores with both low CD4 and CD8 expression were classified as low immune-active, whereas all others were classified as highly immune-active. TC, IC, CPS, and TILs were assessed in all cores, and the highest values among the four cores for NOM, SIN, and HNSCC were recorded. To assess the utility of TC in biopsies, the B7-H4 TC of HNSCC was recorded from both superficial and invasive front cores. The PD-L1 TC was summarized from previous reports; the majority (88%, 65/74) showed positivity in both superficial and invasive front cores [29].

### Construction of TMAs for Visium Spatial Transcriptomics Analysis

Visium ST assays were performed for a total of 18 TMA cores, including six HNSCC, six SIN, and six NOM cores from six patients with progressive HNSCC [sample 1 (S1) to sample 6 (S6)], as previously reported [29]. The clinicopathological characteristics of the patients are presented in Online Resource 2.

### Workflow for the Visium Spatial Transcriptomics Analysis

Visium ST (103 Genomics, Pleasanton, CA, USA) analysis was performed for the spatial gene expression landscape in four annotated clusters: NOM, SIN, HNSCC, and non-evaluable epithelium, which were excluded from the ICI IHC assessment because they lacked nuclei and hemorrhagic area, as previously described [29] and summarized in Online Resource 3. For differential gene expression analysis, the log-fold change threshold was set to 0.25, and *p*-values < 0.05 were adjusted using Bonferroni correction to control for multiple testing. A minimum expression percentage (min.pct) of 0.1 was used to identify significantly differentially expressed genes in each cluster. The significance of mRNA expression was identified as log2 > 0.25 and *p*-value < 0.05, which were adjusted using Bonferroni correction for comparison with other groups in the same cases. Mutually exclusive mRNA expression was defined as *VTCN1*^*+*^ and *CD274*^*−*^*or VTCN1*^*−*^ and *CD274*^*+*^. The log2 fold changes obtained from the differentially expressed genes and up-regulated top 50 genes (log2 > 0.25 and *p*-value < 0.05) were used for GO analysis with ToppGene (https://toppgene.cchmc.org/, accessed: 2024/10/05).

### Statistical Analyses

To measure the mutual exclusivity of B7-H4 and PD-L1 in HNSCC, chi-squared analysis was performed between the mutually exclusive pattern (+) group [B7-H4+/PD-L1- and B7-H4-/PD-L1+) and the (-) group [B7-H4+/PD-L1 + and B7-H4-/PD-L1-]. The concordant expression of B7-H4 TC between the superficial and invasive front cores was examined using the kappa value. Fisher’s exact test was used to determine the correlations between B7-H4 and PD-L1 expression and between B7-H4 expression and clinicopathological features. The e-cutoff value for tumor immune activity was calculated using the area under the curve against overall survival. Statistical analysis was performed using IBM SPSS software (v20.0; IBM Corp., Armonk, NY, USA). Significance was set at *p* < 0.05.

## Results

### B7-H4 and PD-L1 Expression in NOM, SIN, and HNSCC

**Fig. 1:**
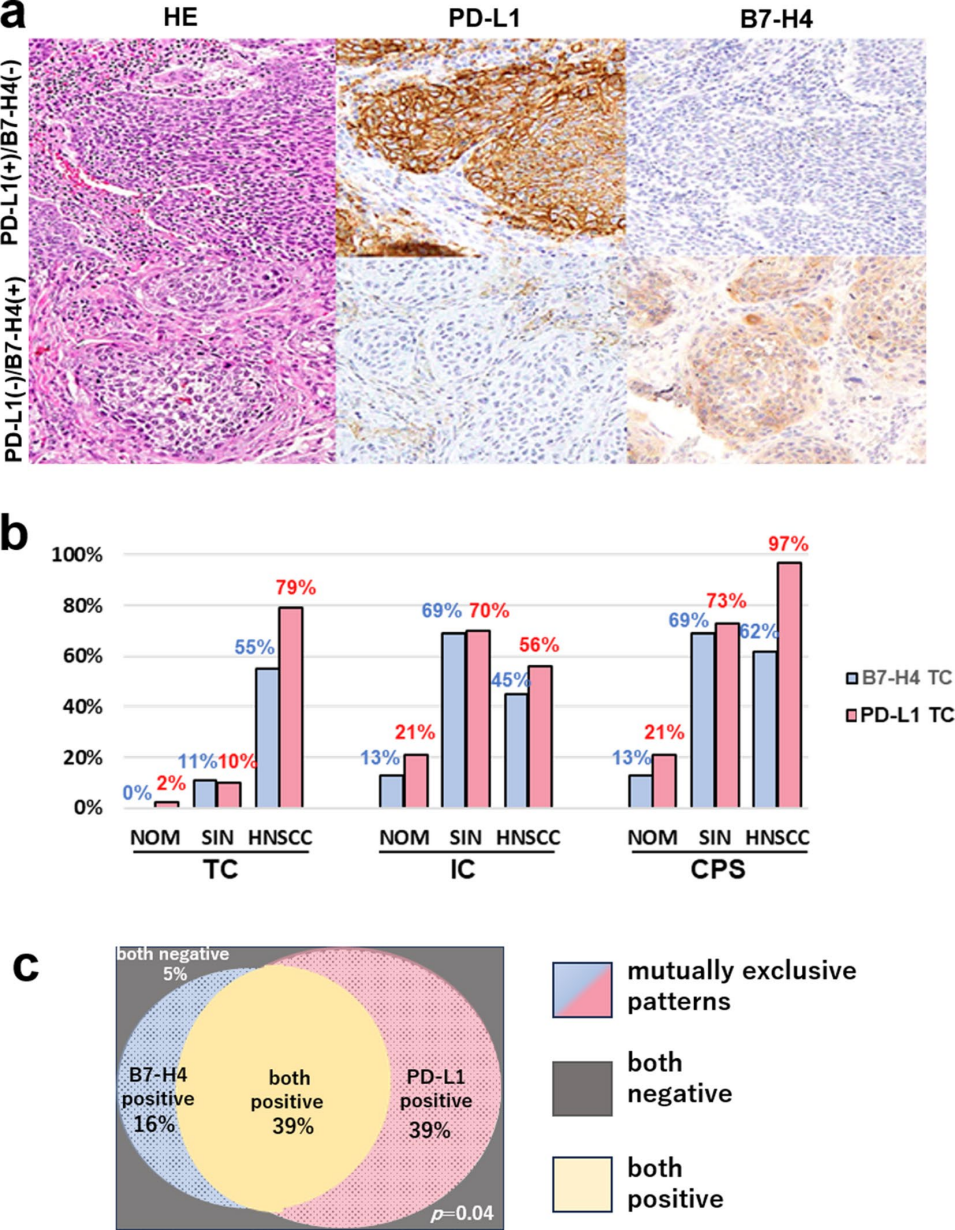
Immunohistochemical staining of B7-H4 and PD-L1 in 94 patients with Hed and Neck Squamous cell carcinomas (**a**) Immunohistochemical expression of PD-L1 (positive, upper row; negative, lower row) and B7-H4 (negative, upper row; positive, lower row. (**b**) Proportion of TC, IC, and CPS positive for B7-H4 and PD-L1 immunohistochemical staining. (**c**) Proportion of B7-H4 staining based on TC in HNSCC (**a**) HE and immunohistochemical staining, original magnification 200×. HE, hematoxylin–eosin; HNSCC, head and neck squamous cell carcinoma; SIN, squamous intraepithelial neoplasm; NOM, normal mucosa; TC, tumor cell score; IC, immune cell score; CPS, combined proportion score; PD-L1, programmed cell death protein-ligand 1

To investigate the potential for a mutually exclusive relationship between B7-H4 and PD-L1 expression in patients with HNSCC, the IHC expression of B7-H4 and PD-L1 was examined using TC, IC, and CPS, which are currently used IHC evaluation methods for PD-L1 expression. The results of the IHC expression analysis for B7-H4 and PD-L1 are presented in Fig. 1a; Table 1.

**Table 1:** Immunohistochemical staining associations for B7-H4 and PD-L1 in HNSCCs

B7-H4 (%, *n* = 94)					
		Negative	Positive	Mutually exclusive patterns*	*p*-value (χ^2^)
PD-L1 (%,)	TC			**55% (52/94)**	**0.04**
	Negative	5% (5/94)	**16% (15/94)**		
	Positive	**39% (37/94)**	39% (37/94)		
	IC			50% (47/94)	0.26
	Negative	31% (29/94)	10% (9/94)		
	Positive	40% (38/94)	19% (18/94)		
	CPS			45% (42/94)	0.37
	Negative	3% (3/94)	7% (7/94)		
	Positive	37% (35/94)	52% (49/94)		

*Mutually exclusive patterns: PD-L1^+^/B7-H4^+^and PD-L1^+^/B7-H4^+^; Bold: *p* < 0.05;TC, tumor cell score; IC, immune cell score CPS; combined proportion score; PD-L1, programmed cell death protein-ligand 1; HNSCC, head and neck squamous cell carcinoma

TC results identified B7-H4^+^ and PD-L1^+^ most frequently in HNSCC and almost no NOM or SIN (B7-H4: NOM: 0%, SIN: 1%, and HNSCC: 55% and PD-L1: NOM: 2%, SIN: 10%, and HNSCC: 79%; Fig. 1b) with a significant mutually exclusive expression pattern between B7-H4 and PD-L1 in patients with HNSCC (55%, 52/94, *p* = 0.04: Fig. 1a-c; Table 1). Conversely, the IC and CPS results identified B7-H4^+^ and PD-L1^+^ in HNSCC, as well aso NOM and SIN; IC (B7-H4: NOM: 13%, SIN: 69%, and HNSCC: 45%, and PD-L1: NOM: 21%, SIN: 70%, and HNSCC: 56%) and CPS (NOM: 13%, SIN: 69%, and HNSCC: 62%, and PD-L1: NOM: 21%, SIN: 73%, and HNSCC: 97%). Moreover, no significant mutually exclusive expression pattern between B7-H4 and PD-L1 in HNSCC cases was observed with IC (50%, 47/94, *p* = 0.26) and CPS (45%, 42/94, *p* = 0.37). Between the superficial and deep area cores of OSCC, concordant B7-H4 expression was observed in 95% (90/94) of cases, with a kappa value of 0.91 (95% CI; 0.83–0.99), indicating a near match. Based on these findings, TC was selected as the most suitable method for evaluating B7-H4.

### Clinicopathological Analysis of B7-H4 Expression

To examine the association of B7-H4, as an ADC of PD-L1 negative HNSCC, with tumor immunity, we examined the association between B7-H4 expression and the clinicopathological features of 94 patients with progressive HNSCC (Table 2). B7-H4^+^ expression was significantly associated with low CD8^+^ lymphocytes infiltration; 34% of B7-H4(-) vs. 66% of B7-H4^+^ in low CD8 and 64% B7-H4^−^ vs. 36% of B7-H4^+^ in high CD8 (*p* = 0.009, Table 2). No associations were detected in other clinicopathological features, including CD4^+^ lymphocyte infiltration, immune-reactive TILs, and tumor microenvironmental pathological features.

**Table 2:** Clinicopathological analysis of B7-H4 expression

		B7-H4 expression (HNSCC, *n* = 94)		
Clinicopathological features		Negative (TC < 1%)	Positive (TC ≥ 1%)	*p*-value
Differentiation				0.68
	Well or moderately	23 (43%)	31 (57%)	
	Poorly	19 (48%)	21 (53%)	
**Ly**				0.61
	Negative	7 (37%)	12 (63%)	
	Positive	35 (100%)	0 (0%)	
V				0.57
	Negative	8 (53%)	7 (47%)	
	Positive	34 (100%)	0 (0%)	
Pn				0.80
	Negative	8 (40%)	12 (60%)	
	Positive	34 (46%)	40 (54%)	
Invasion pattern				0.29
	YK-1,2	6 (67%)	3 (%33)	
	YK-3,4	36 (42%)	49 (58%)	
pDOI				0.50
	< 10 mm	3 (30%)	7 (70%)	
	≥ 10 mm	39 (46%)	45 (54%)	
Lymph node metastasis				> 0.999
	Presence	20 (44%)	25 (56%)	
	Absence	22 (45%)	27 (55%)	
pENE				0.82
	Absence	30 (43%)	39 (57%)	
	Presence	12 (48%)	13 (52%)	
BUD				0.53
	Low	20 (49%)	21 (51%)	
	High	22 (42%)	31 (58%)	
DR				> 0.999
	Mature	11 (46%)	13 (54%)	
	Immature	31 (44%)	39 (56%)	
TILs				0.28
	Low	25 (40%)	37 (60%)	
	High	17 (53%)	15 (47%)	
CD4				0.21
	Low	20 (38%)	32 (62%)	
	High	22 (52%)	20 (48%)	
**CD8**				***0.009***
	Low	21 (34%)	**40 (66%)**	
	High	**21 (64%)**	12 (36%)	
Immune-active				0.2
	Low	15 (38%)	24 (62%)	
	High	27 (49%)	28 (51%)	

Bold: *p* < 0.05; BUD, tumor budding; DR, desmoplastic reaction; HNSCC, head and neck squamous cell carcinoma; Ly, lymphovascular invasion; pDOI, pathological depth of invasion; pENE, pathological extranodal extension; Pn, perineural invasion; TIL, tumor-infiltrating lymphocyte; V, vascular invasion; TC, tumor cell score

### Associations of B7-H4 and PD-L1 Expression and mRNA Distributions in HNSCC Tissues

**Fig. 2:**
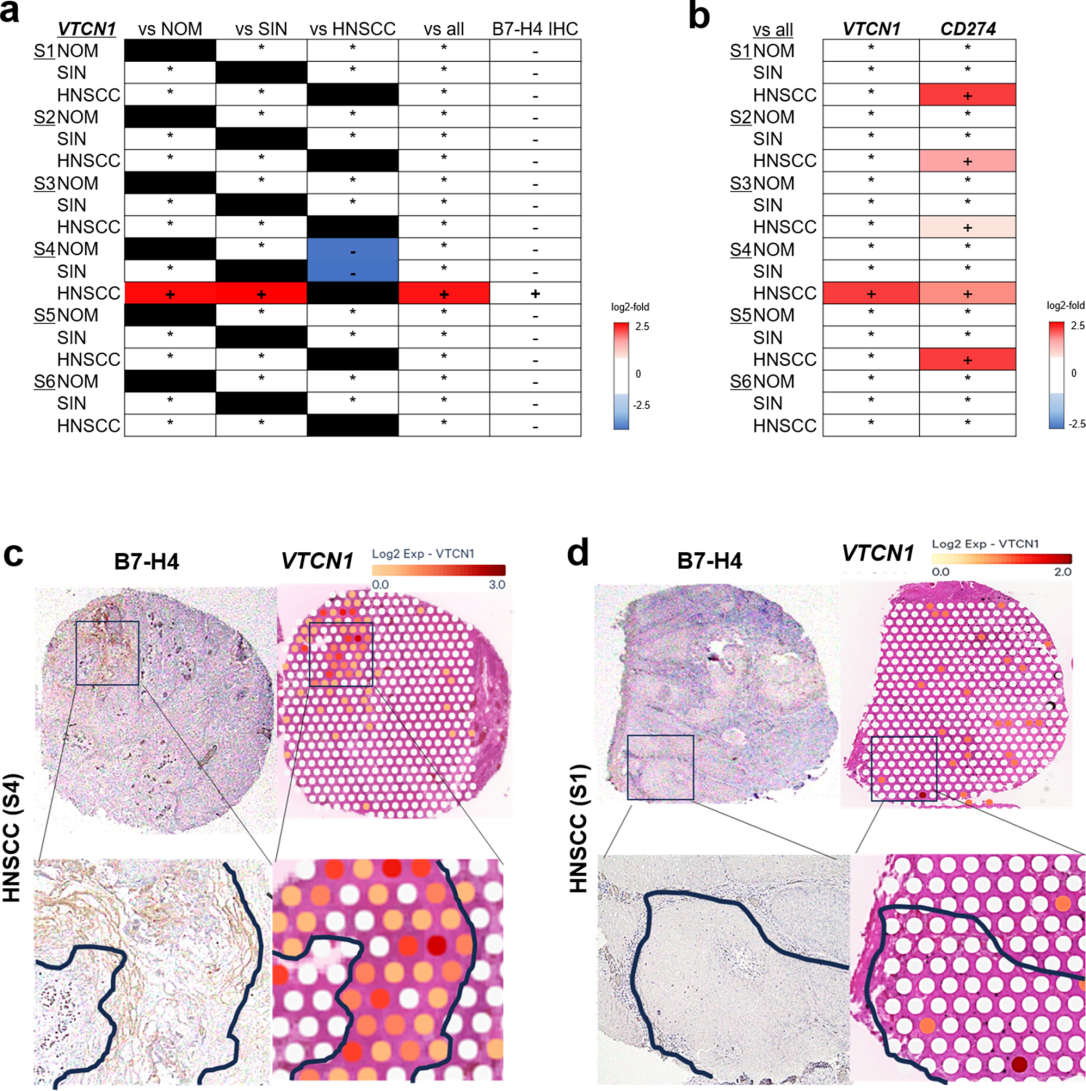
Spatial transcriptomic distribution and expression of B7-H4, *VTCN1*, and *CD274.* (**a**) *VTCN1* and associated protein-mRNA expression and (**b**) *VTCN1* and *CD274* expression in 18 tissue cores (six HNSCC cores, six SIN cores, and six NOM cores) obtained from six patients with HNSCC. (**b-d**) Matched distributions of the immunohistochemical expression areas (left) and mRNA upregulated areas (right) for B7-H4 (*VTCN1*). (**c**) *VTCN1* overexpression and (**d**) non-overexpression areas. S, sample; HNSCC, head and neck squamous cell carcinoma; SIN, squamous intraepithelial neoplasm; NOM, normal mucosa; vs. NOM, compared to the NOM; vs. SIN, compared to the SIN; vs. HNSCC, compared to the HNSCC; vs. all, compared among NOM, SIN, and HNSCC

Eighteen tissue cores (six HNSCC cores, six SIN cores, and six NOM cores) obtained from six patients with HNSCC were analyzed using spatial transcriptomics. The results revealed the associated protein-mRNA expression and distribution; 100% (18/18) had B7-H4 and *VTCN1* expression in the epithelium. B7-H4^+^ and *VTCN1*^+^ (significant *VTCN1* upregulation among the NOM, SIN, and HNSCC cores from the same patient) were detected in one HNSCC core (S4) (Fig. 2a, b, and Online Resources 3–5). None of the remaining 17 cores expressed B7-H4 or *VTCN1*. In contrast to *VTCN1*, significant *CD274* upregulation was detected in five HNSCC cores (S1-5) (Fig. 2b and Online Resource 3–5).

**Fig. 3:**
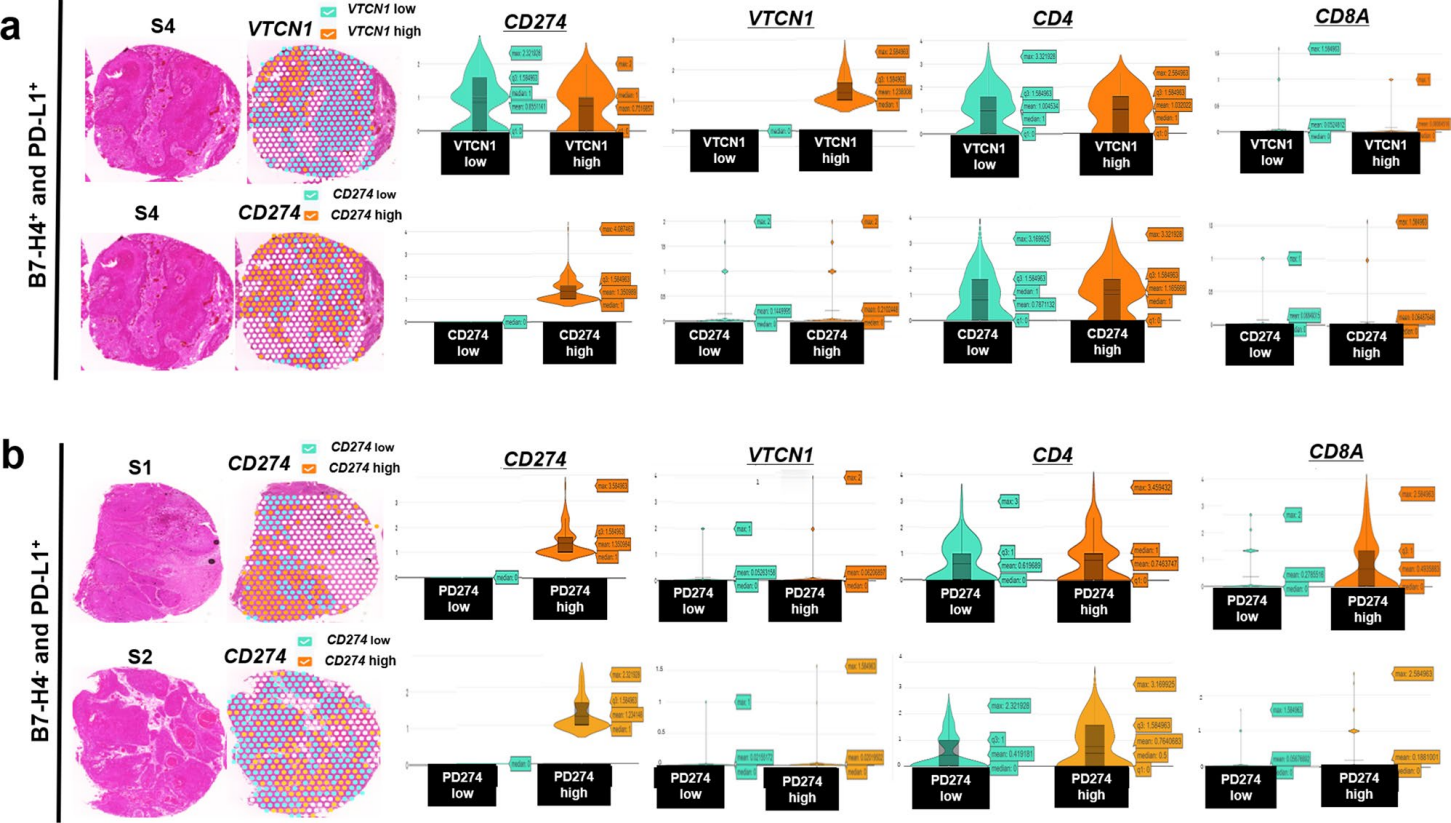
Spatial transcriptomic distribution and expression of B7-H4 (*VTCN1*), PD-L1 (*CD274*), *CD4*, and *CD8A* (**a**,** b**) *CD274*,* VTCN1*, *CD4*, and *CD8A* expression levels compared with *CD274* and *VTCN1* overexpression and non-overexpression areas. S, sample; HNSCC, head and neck squamous cell carcinoma; PD-L1, programmed cell death protein-ligand 1

The expression and distribution of *VTCN1* and *CD274* were examined in the HNSCC cores. One HNSCC core (S4; positive for *VTCN1*^+^ and *CD274*^*+*^) showed the same expression levels in the *VTCN1*^+^ and *VTCN1*^−^ areas (Fig. 3a, log2 median expression value = 1). The five HNSCC cores with *CD274*^+^ exhibited lower *VTCN1* expression in the *CD274*^+^ area than in the *CD274*^−^ area (Fig. 3b and Online Resource 5). Thus, we detected mutually exclusive expression and distribution of B7-H4 (*VTCN1)* and PD-L1 (*CD274)* in five cases (83%, 5/6), whereas one HNSCC (S4) core was double-positive for B7-H4 (*VTCN1*) and PD-L1 (*CD274*).

### Associations of B7-H4 and PD-L1 with the Tumor Immune-Response in HNSCC Tissues

Based on the clinicopathological negative association of B7-H4 expression and CD8^+^ TILs, the characteristics of *VTCN1* in the tumor immune-response of HNSCC were further analyzed. *CD8A* was downregulated in the *VTCN1*^+^ area compared with expression in the *VTCN1*^−^ area (S4), whereas *CD8A* was upregulated in the *CD274*^+^ area compared with expression in the *CD274*^−^ area (S1-6, Fig. 3a, b, and Online Resource 5). No other differences in *CD4* expression were identified in the *CD274*^+ or −^ and *VTCN1*^+ or–^ areas (Fig. 3a, b, and Online Resource 5). This indicated that *CD8A*-positive T-cell infiltration may be suppressed within *VTCN1*^+^ area of the HNSCC epithelium.

Pathway analysis was not performed because the mechanisms of B7-H4 expression are not well understood. For a detailed examination of the tumor immunological function of *VTCN1*, further GO analysis was performed using the top 50 genes upregulated in the *VTCN1*^*+*^ area compared with those in the *VTCN1*^*−*^ area, but only mRNAs associated with cellular homeostasis regulatory mechanisms and not those associated with the immune-response were collected (Online Resource 6).

## Discussion

This study demonstrated that TC is the preferred IHC evaluation method for the mutually exclusive expression patterns of B7-H4 and PD-L1 in patients with HNSCC. These mutually exclusive expression distributions were observed in over half the HNSCC cases examined at both the protein and mRNA levels. Furthermore, clinicopathological analysis revealed a negative correlation between B7-H4 expression and CD8^+^ lymphocytes, which was supported by the mRNA data. The data were collected from small TMA cores, indicating that B7-H4 TC can be readily applied to small specimens, such as biopsies, which are associated with tumor immunity. This indicates that B7-H4 as a novel ADC target may be a promising approach, especially for patients in whom PD-L1 is less predictive.

B7-H4, which is overexpressed in immune-desert tumors with low or absent PD-L1 expression, has garnered attention in malignant solid tumors [11, 14–16]. There is only one IHC study on HNSCC, which found a positive association between tumoral B7-H4 and PD-L1 [33]; however, the low correlation coefficient and computerized evaluation system may explain their conflicting results. In this study, we examined the most suitable IHC evaluation method, among those currently used for ICIs, for B7-H4 IHC associated with HNSCC. TC exhibited a mutually exclusive IHC pattern between B7-H4 and PD-L1 in over half of the HNSCC cases. TC has a slightly lower expression level than PD-L1 CPS assays [34], and it provides more insights into the tumor’s immune evasion mechanisms than other ICI analysis methods [35]. This result was consistent with the immunohistochemical and clinicopathological data obtained from our analysis. Notably, a high concordance expression of TC > 1% was found between superficial and invasive front cores, even when using small TMA cores comparable to biopsies, and the frequency ranges for B7-H4 and PD-L1 in this study were consistent with previous findings [15, 22, 25, 27, 36]. The ability to detect exclusive expressions highlights the suitability of TCs for B7-H4 analysis.

This study also revealed that IHC expression of B7-H4 corresponds with its mRNA distribution. Previous studies reported the association of B7-H4 and PD-L1 IHC with mRNA expression; however, their protein and mRNA distributions and spatial transcriptomic data have not been reported. This study is the first to elucidate these data at the single-cell level. Due to time limitations in sample collection, this study did not include mRNA expression data from the B7-H4 ^+^/PD-L1– cases in the ST analysis. However, we did identify a multimodal pattern encompassing B7-H4 (*VTCN1*) ^−^/PD-L1 (*CD274*) ^+^, co-expressed, and double-negative expression with matched protein-mRNA expression and distribution. Considering the concordance of protein-mRNA expression and that over half of the HNSCCs showed a mutually exclusive B7-H4/PD-L1 pattern in the TMA IHC analysis, HNSCC cases with B7-H4^+^/PD-L1– may correspond with mRNA expression and express mutually exclusive patterns at the mRNA level.

Furthermore, ST analysis also indicated the novel potential of a therapeutic standpoint. Although this study did not detect specific molecular functions and biological processes related to B7-H4 expression, we identified a multimodal pattern for B7-H4/PD-L1. B7-H4 is a promising selective drug target in patients with HNSCC and low PD-L1 expression who are ineligible for ICI treatment. Although the mechanisms underlying B7-H4 expression remain unclear, B7-H4 protein expression is limited in normal tissue but markedly overexpressed in several cancers, including HNSCC, and this is supported by both the existing literature and this study [12, 13]. Given that B7-H4 inhibits immune activation even after anti-PD-L1 treatment [37, 38], the current finding, a multimodal pattern for B7-H4/PD-L1, indicates that PD-L1 and B7-H4 may be independently regulated, and thus, dual blockades of PD-L1 and B7-H4 could enhance therapeutic efficacy [12].

With respect to the role of B7-H4 in anti-tumor immunity, clinicopathological and ST analyses indicate that it has the potential to suppress CD8^+^ NKT cell-mediated anti-tumor responses. Although the expression of B7-H4 in T cells is currently unclear, tumors can evade the immune response via B7-H4 engagement with their T cell receptors, thereby inhibiting the proliferation of CD4^+^ and CD8^+^ T cells [12, 13, 39]. Our findings are consistent with the proposed mechanism. The absence of a significant association with CD4^+^ TILs may be due to variations in the evaluation criteria, organs, and tumor types. Given the established association between low CD8 + TIL infiltration and poor prognosis in HNSCC, regardless of HPV status [40, 41], B7-H4 represents a promising novel target for ADC development. In fact, several ADCs designed to block B7-H4–mediated T cell activity are currently under development [12]. For example, a phase I/IIa trial of the B7-H4-targeting ADC AZD8205 (NCT05123482) is underway for progressive solid malignancies, including HNSCC.

This study has some limitations. First, this was a single-institute study with a limited sample size, and data after ICI treatment were not included. Second, although the PD-L1 clone 73 − 10 is not a companion diagnostic for HNSCC, it has greater PD-L1 detection sensitivity than other clones [25–27, 29]. It could indicate that more large number of patients could be eligible for B7-H4-targeted therapy than that with HNSCC who 73 − 10 detected PD-L1- expression. Third, because the *VTCN1* expression pathway is not well established and a limited number of *VTCN1*^*+*^ cores were obtained using spatial transcriptomics, we could not investigate the expression mechanism of B7-H4 in patients with HNSCC. Fourth, the association between the TC for B7-H4 and HNSCC outcomes was not shown due to the limited number of patients with HNSCCs who were 73 − 10 detected PD-L1-negative. However, B7-H4 is a negative prognostic marker in several malignant tumors [12]. Therefore, further investigation is required to validate the evaluation method, immunological mechanisms, treatment response, and prognostic significance of B7-H4 expression, considering the results of ongoing phase I/IIa studies.

## Conclusion

B7-H4 expression in tumor cells is a potential novel target for ADC development in ICI-resistant progressive HNSCC. The possible mechanism of action is the suppression of CD8^+^ T cell infiltration. The results showed that IHC TC can provide an accurate and reliable assessment of B7-H4 expression, even in small, limited biopsy-like samples, making it a clinically feasible approach.

## Electronic Supplementary Material

Below is the link to the electronic supplementary material.

Supplementary Material 1: Online Resource 1. Summary of patient characteristics

Supplementary Material 2: Online Resource 2. Summary of patient characteristics

Supplementary Material 3: Online Resource 3. Differential expression analysis and the association between immunohistochemical staining and the Visium annotation area

Supplementary Material 4: Online Resource 4. Visium DEGs among the three groups

Supplementary Material 5: Online Resource 5. Protein and mRNA expression data obtained via spatial analysis from PD-L1 positive cases (S1-6) and B7-H4 positive cases (S4)

Supplementary Material 6: Online Resource 6. Gene ontology (GO) analysis summary

## Data Availability

No datasets were generated or analysed during the current study.

## References

[1] Barsouk A, Aluru JS, Rawla P, Saginala K, Barsouk A (2023) Epidemiology, risk factors, and prevention of head and neck squamous cell carcinoma. Med Sci 11:42. 10.3390/medsci11020042 10.3390/medsci11020042 PMC10304137 37367741

[2] Proulx-Rocray F, Soulières D (2024) Emerging monoclonal antibody therapy for head and neck squamous cell carcinoma. Expert Opin Emerg Drugs 29:165–176. 10.1080/14728214.2024.2339906 38616696 10.1080/14728214.2024.2339906

[3] Osazuwa-Peters N et al (2018) Suicide risk among cancer survivors: head and neck versus other cancers. Cancer 124:4072–4079. 10.1002/cncr.31675 30335190 10.1002/cncr.31675

[4] Chow LQM, Longo DL (2020) Head and neck cancer. N Engl J Med 382:60–72 31893516 10.1056/NEJMra1715715

[5] Chen Y, Ding X, Bai X et al (2023) The current advances and future directions of PD-1/PD-L1 Blockade in head and neck squamous cell carcinoma (HNSCC) in the era of immunotherapy. Int Immunopharmacol 120:110329. 10.1016/j.intimp.2023.110329 37207445 10.1016/j.intimp.2023.110329

[6] Parmar K, Mohamed A, Vaish E, Thawani R, Cetnar J, Thein KZ (2022) Immunotherapy in head and neck squamous cell carcinoma: an updated review. Cancer Treat Res Commun 33:100649. 10.1016/j.ctarc.2022.100649 36279709 10.1016/j.ctarc.2022.100649

[7] Ferris RL, Blumenschein G, Fayette J et al (2016) Nivolumab for recurrent squamous-cell carcinoma of the head and neck. N Engl J Med 375:1856–1867. 10.1056/NEJMoa1602252 27718784 10.1056/NEJMoa1602252 PMC5564292

[8] Chalker C, Voutsinas JM, Wu QV et al (2022) Performance status (PS) as a predictor of poor response to immune checkpoint inhibitors (ICI) in recurrent/metastatic head and neck cancer (RMHNSCC) patients. Cancer Med 11:4104–4111. 10.1002/cam4.4722 35349227 10.1002/cam4.4722 PMC9678089

[9] Nocini R, Vianini M, Girolami I et al (2022) PD-L1 in oral squamous cell carcinoma: A key biomarker from the laboratory to the bedside. Clin Exp Dent Res 8:690–698. 10.1002/cre2.590 35593124 10.1002/cre2.590 PMC9209791

[10] Emancipator K, Huang L, Aurora-Garg D et al (2021) Comparing programmed death ligand 1 scores for predicting pembrolizumab efficacy in head and neck cancer. Mod Pathol 34:532–541. 10.1038/s41379-020-00710-9 33239737 10.1038/s41379-020-00710-9

[11] Ribbat-Idel J, Dressler FF, Krupar R et al (2021) Performance of different diagnostic PD-L1 clones in head and neck squamous cell carcinoma. Front Med 8:640515. 10.3389/fmed.2021.640515 10.3389/fmed.2021.640515 PMC8110724 33987192

[12] MacGregor HL, Ohashi PS (2017) Molecular pathways: evaluating the potential for B7-H4 as an immunoregulatory target. Clin Cancer Res 23:2934–2941. 10.1158/1078-0432 28325750 10.1158/1078-0432.CCR-15-2440

[13] Xiao L, Guan X, Xiang M, Wang Q, Long Q, Yue C, Chen L, Liu J, Liao C (2022) B7 family protein glycosylation: promising novel targets in tumor treatment. Front Immunol 13:1088560. 10.3389/fimmu.2022.1088560 36561746 10.3389/fimmu.2022.1088560 PMC9763287

[14] Spranger S, Bao R, Gajewski TF (2015) Melanoma-intrinsic β-catenin signalling prevents anti-tumour immunity. Nature 523:231–235. 10.1038/nature14404 25970248 10.1038/nature14404

[15] Chen D, Li G, Ji C et al (2020) Enhanced B7-H4 expression in gliomas with low PD-L1 expression identifies super-cold tumors. J Immunother Cancer 8:e000154. 10.1136/jitc-2019-000154 32457124 10.1136/jitc-2019-000154 PMC7253052

[16] Bonaventura P, Shekarian T, Alcazer V et al (2019) Cold tumors: a therapeutic challenge for immunotherapy. Front Immunol 10:168. 10.3389/fimmu.2019.00168 30800125 10.3389/fimmu.2019.00168 PMC6376112

[17] Song X, Zhou Z, Li H et al (2020) Pharmacologic suppression of B7-H4 glycosylation restores antitumor immunity in immune-cold breast cancers. Cancer Discov 10:1872–1893. 10.1158/2159-8290.CD-20-0402 32938586 10.1158/2159-8290.CD-20-0402 PMC7710601

[18] Sousa LG, McGrail DJ, Lazar Neto F et al (2023) Spatial Immunoprofiling of adenoid cystic carcinoma reveals B7-H4 is a therapeutic target for aggressive tumors. Clin Cancer Res 29:3162–3171. 10.1158/1078-0432.CCR-23-0514 37256648 10.1158/1078-0432.CCR-23-0514 PMC10526680

[19] Yan X, Hong B, Feng J et al (2022) B7-H4 is a potential diagnostic and prognostic biomarker in colorectal cancer and correlates with the epithelial-mesenchymal transition. BMC Cancer 22:1053. 10.1186/s12885-022-10159-5 36217128 10.1186/s12885-022-10159-5 PMC9549643

[20] Schalper KA, Carvajal-Hausdorf D, McLaughlin J et al (2017) Differential expression and significance of PD-L1, IDO-1, and B7-H4 in human lung cancer. Clin Cancer Res 23:370–378. 10.1158/1078-0432.CCR-16-0150 27440266 10.1158/1078-0432.CCR-16-0150 PMC6350535

[21] Yang J, Tian Z, Gao H et al (2022) Clinical significance and correlation of PD-L1, B7-H3, B7-H4, and TILs in pancreatic cancer. BMC Cancer 22:584. 10.1186/s12885-022-09639-5 35624419 10.1186/s12885-022-09639-5 PMC9137118

[22] Chen L, Dong J, Li Z, Chen Y, Zhang Y (2022) The B7H4-PDL1 classifier stratifies immuno-phenotype in cervical cancer. Cancer Cell Int 22:3. 10.1186/s12935-021-02423-8 34983532 10.1186/s12935-021-02423-8 PMC8728907

[23] Kinneer K, Wortmann P, Cooper ZA et al (2023) Design and preclinical evaluation of a novel B7-H4-directed antibody-drug conjugate, AZD8205, alone and in combination with the PARP1-selective inhibitor AZD5305. Clin Cancer Res 29:1086–1101. 10.1158/1078-0432.CCR-22-2630 36355054 10.1158/1078-0432.CCR-22-2630

[24] Toader D, Fessler SP, Collins SD et al (2023) Discovery and preclinical characterization of XMT-1660, an optimized B7-H4-targeted antibody-drug conjugate for the treatment of cancer. Mol Cancer Ther 22:999–1012. 10.1158/1535-7163.MCT-22-0786 37294948 10.1158/1535-7163.MCT-22-0786 PMC10477829

[25] Ikeda J, Ohe C, Yoshida T et al (2021) PD-L1 expression and clinicopathological factors in renal cell carcinoma: a comparison of antibody clone 73– 10 with clone 28– 8. Anticancer Res 41:4577–4586. 10.21873/anticanres.15271 34475086 10.21873/anticanres.15271

[26] Tsao MS, Kerr KM, Kockx M et al (2018) PD-L1 immunohistochemistry comparability study in real-life clinical samples: results of blueprint phase 2 project. J Thorac Oncol 13:1302–1311. 10.1016/j.jtho.2018.05.013 29800747 10.1016/j.jtho.2018.05.013 PMC8386299

[27] Grote HJ, Feng Z, Schlichting M et al (2020) Programmed death-ligand 1 immunohistochemistry assay comparison studies in NSCLC: characterization of the 73– 10 assay. J Thorac Oncol 15:1306–1316. 10.1016/j.jtho.2020.04.013 32353599 10.1016/j.jtho.2020.04.013

[28] Ståhl PL, Salmén F, Vickovic S, Lundmark A, Navarro JF, Magnusson J, Giacomello S, Asp M, Westholm JO, Huss M, Mollbrink A, Linnarsson S, Codeluppi S, Borg Å, Pontén F, Costea PI, Sahlén P, Mulder J, Bergmann O, Lundeberg J, Frisén J (2016) Visualization and analysis of gene expression in tissue sections by Spatial transcriptomics. Science 353:78–82. 10.1126/science.aaf2403 27365449 10.1126/science.aaf2403

[29] Noda Y, Atsumi N, Nakaya T, Iwai H, Tsuta K (2025) High-sensitivity PD-L1 staining using clone antibody and spatial transcriptomics for precise expression analysis in non-tumorous, intraepithelial neoplasia, and squamous cell carcinoma of head and neck. Head Neck Pathol Submitted for publication 73– 10 10.1007/s12105-025-01798-8 PMC12092928 40392349

[30] Akhtar M, Rashid S, Al-Bozom IA (2021) PD-L1 immunostaining: what pathologists need to know. Diagn Pathol 16:94. 10.1186/s13000-021-01151-x 34689789 10.1186/s13000-021-01151-x PMC8543866

[31] Blatt S, Krüger M, Rump C, Zimmer S, Sagheb K, Künzel J (2022) Differences in PD-L1 expression between oral and oropharyngeal squamous cell carcinoma. PLoS ONE 17:e0269136. 10.1371/journal.pone.0269136 35622885 10.1371/journal.pone.0269136 PMC9140279

[32] Jeong JS, Jo U, Choi G, Song H, Cho KJ, Song JS (2024) Comparison of PD-L1 assays in head and neck carcinoma. Pathology 56:969–981. 10.1016/j.pathol.2024.06.006 39261273 10.1016/j.pathol.2024.06.006

[33] Wu L, Deng WW, Yu GT et al (2016) B7-H4 expression indicates poor prognosis of oral squamous cell carcinoma. Cancer Immunol Immunother 65:1035–1045. 10.1007/s00262-016-18 27383830 10.1007/s00262-016-1867-9 PMC11029220

[34] Ito T, Okamoto I, Tokashiki K, Okada T, Yamashita G, Nagao T, Hirai H, Saigusa N, Tsukahara K (2022) PD-L1 expression and survival rates using TPS and CPS for nivolumab-treated head-and-neck cancer. Anticancer Res 42(3):1547–1554 35220251 10.21873/anticanres.15628

[35] Wildsmith S, Ye J, Franks A et al (2022) Association of PD-L1 expression on tumor and immune cells with survival in recurrent or metastatic head and neck squamous cell carcinoma and assay validation. Cancer Res Commun 2(1):39–48 Published 2022 Jan 20. 10.1158/2767-9764.CRC-21-0032 36860696 10.1158/2767-9764.CRC-21-0032 PMC9973403

[36] Zong L, Gu Y, Zhou Y et al (2022) Expression of B7 family checkpoint proteins in cervical cancer. Mod Pathol 35:786–793. 10.1038/s41379-021-00979-4 34848831 10.1038/s41379-021-00979-4

[37] Liu Y, John P, Nishitani K et al (2023) A SOX9-B7x axis safeguards dedifferentiated tumor cells from immune surveillance to drive breast cancer progression. Dev Cell 58:2700–2717e12. 10.1016/j.devcel.2023.10.01 37963469 10.1016/j.devcel.2023.10.010 PMC10842074

[38] Wescott EC, Sun X, Gonzalez-Ericsson P et al (2024) Epithelial expressed B7-H4 drives differential immunotherapy response in murine and human breast cancer. Cancer Res Commun 4:1120–1134. 10.1158/2767-9764.CRC-23-0468 38687247 10.1158/2767-9764.CRC-23-0468 PMC11041871

[39] Altan M, Pelekanou V, Schalper KA et al (2017) B7-H3 expression in NSCLC and its association with B7-H4, PD-L1 and tumor-infiltrating lymphocytes. Clin Cancer Res 23:5202–5209. 10.1158/1078-0432.CCR-16-3107 28539467 10.1158/1078-0432.CCR-16-3107 PMC5581684

[40] Borsetto D, Tomasoni M, Payne K, Polesel J, Deganello A, Bossi P, Tysome JR, Masterson L, Tirelli G, Tofanelli M, Boscolo-Rizzo P (2021) Prognostic significance of CD4 + and CD8 + tumor-infiltrating lymphocytes in head and neck squamous cell carcinoma: A meta-analysis. Cancers 13:781. 10.3390/cancers13040781 33668519 10.3390/cancers13040781 PMC7918220

[41] Stasikowska-Kanicka O, Wągrowska-Danilewicz M, Danilewicz M (2018) Immunohistochemical analysis of Foxp3+, CD4+, CD8 + cell infiltrates and PD-L1 in oral squamous cell carcinoma. Pathol Oncol Res 24(3):497–505. 10.1007/s12253-017-0270-y 28669079 10.1007/s12253-017-0270-y PMC5972165

