# Supplementary material for: Spatial Transcriptome Analysis of B7-H4 in Head and Neck Squamous Cell Carcinoma: A Novel Therapeutic Target for Anti-Immune Checkpoint Inhibitors
Source: Head Neck Pathol. 2025 Jun 30;19(1):78. doi: 10.1007/s12105-025-01815-w (PMC12209170; doi:10.1007/s12105-025-01815-w)
Supplement: Supplementary file 2 — Supplementary Material 2: Online Resource 2. Summary of patient characteristics [file 12105_2025_1815_MOESM2_ESM.docx]

**Online Resource 2.** Summary of patient characteristics

| Patient characteristics | n = 94 |
| --- | --- |
| Age |  |
| Range | 30–87 |
| Median | 69 |
| Average | 68.7±10.9 |
| Sex |  |
| Male:female | 59:36 |
| Location |  |
| Buccal mucosa | 6 |
| Gingiva | 26 |
| Floor of the mouth | 1 |
| Tongue | 61 |
| pT |  |
| 3.4 | 94 |
| TILs |  |
| Low | 62 |
| High | 32 |
| CD4(+) T-cell infiltration |  |
| Low | 52 |
| High | 42 |
| CD8(+) T-cell infiltration |  |
| Low | 61 |
| High | 33 |
| Immunophenotype |  |
| Hot | 74 |
| Desert | 20 |
| Lymph node metastasis | |
| Absence (pN0) | 45 |
| Presence (pN1-3) | 49 |
| Recurrence |  |
| Absence | 64 |
| Presence | 30 |
| Death |  |
| Absence | 54 |
| Presence (HNSCC-related) | 41 (28) |
| Survival time (months) |  |
| Range | 1–180 |
| Median | 42 |
| Average | 47.7±39.9 |

TILs, tumor infiltrating lymphocytes; p, pathological; HNSCC, head and neck squamous cell carcinoma; lymphovascular invasion.
