# Supplementary material for: Spatial Transcriptome Analysis of B7-H4 in Head and Neck Squamous Cell Carcinoma: A Novel Therapeutic Target for Anti-Immune Checkpoint Inhibitors
Source: Head Neck Pathol. 2025 Jun 30;19(1):78. doi: 10.1007/s12105-025-01815-w (PMC12209170; doi:10.1007/s12105-025-01815-w)
Supplement: Supplementary file 3 — Supplementary Material 3: Online Resource 3. Differential expression analysis and the association between immunohistochemical staining and the Visium annotation area [file 12105_2025_1815_MOESM3_ESM.docx]

**Online Resource 3.** Differential expression analysis and the association between immunohistochemical staining and the Visium annotation area


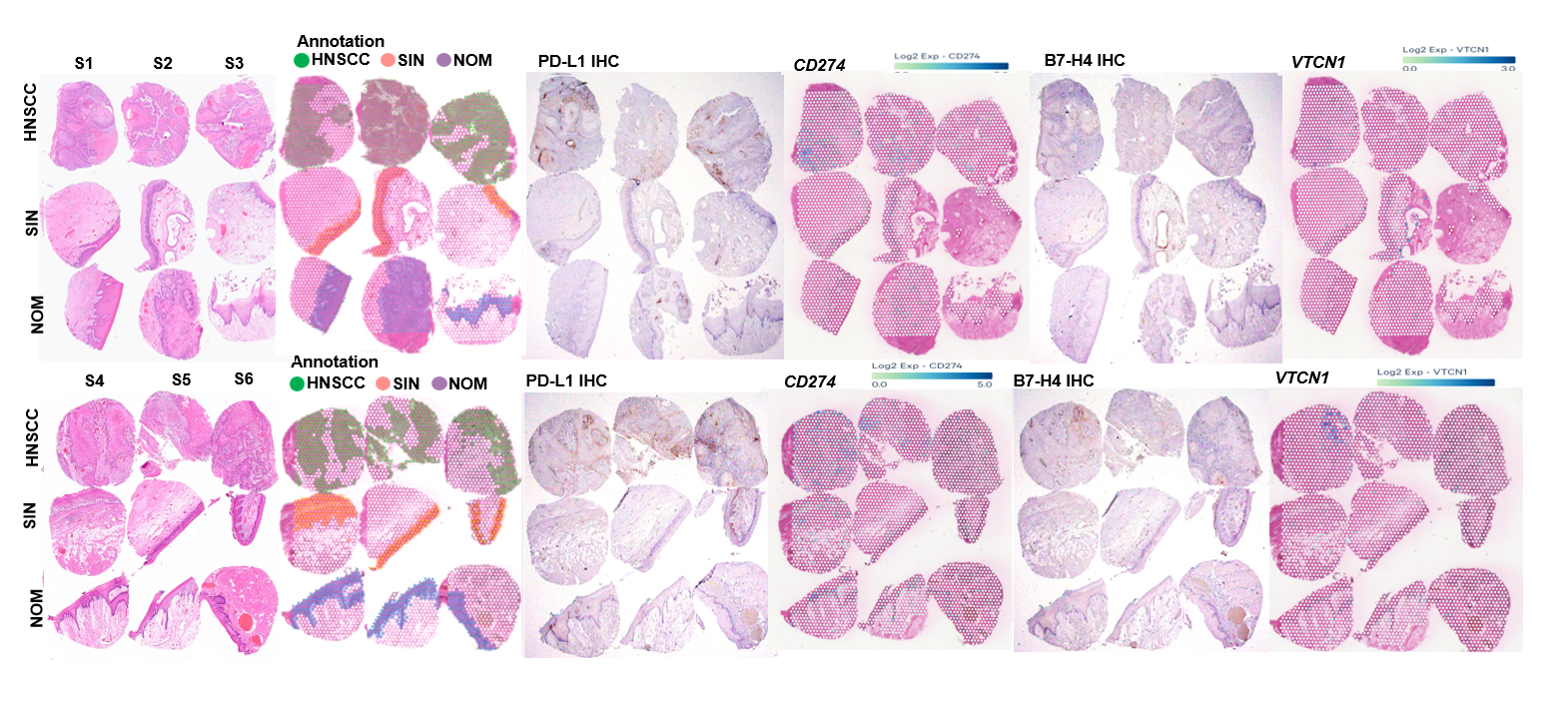


Association and distribution of the protein and mRNA expression of PD-L1 (*CD274*) and B7-H4 (*VTCN1*) in HNSCC tissue sections of NOM, SIN, and HNSCC. The protein and mRNA distributions of PD-L1 (*CD274*) and B7-H4 (*VTCN1*) are matched and show mutually exclusive patterns (arrows). IHC, immunohistochemistry; NOM, normal; SIN, squamous intraepithelial neoplasia; HNSCC, head and neck squamous cell carcinoma;; TC, tumor cell score; S, sample

**Differential Expression Analysis**

In this study, differential gene expression analysis was conducted using scRNA-seq datasets to compare expression patterns between six different samples (S1 to S6) and three clusters (Epithelium areas of NOM, SIN, and HNSCC). The analysis was performed utilizing the Seurat package (v4.1.1) as follows;

**1. Data Preprocessing**

Quality control measures were applied to remove low-quality cells based on the following thresholds:

- **UMI count**: Cells with fewer than 500 unique molecular identifiers (UMIs) were excluded.
- **Mitochondrial gene content (percent.mt)**: Cells were excluded if the mitochondrial gene content exceeded the following sample-specific thresholds: S1: 5%, S2: 5% , S3: 5%, S4: 4%, S5: 4%, S6: 5%.
- **Gene count (nFeature_RNA) and total RNA count (nCount_RNA)**: Cells were filtered using the following criteria:　**S1, S2, and S3** (nFeature_RNA: 500 to 9,000 and nCount_RNA: Less than 50,000), and **S4, S5, and S6** (nFeature_RNA: 300 to 8,000 and nCount_RNA: Less than 40,000).

**2. Differential Gene Expression Analysis**

After t**ranscriptomics data processing, d**ifferential gene expression analysis between clusters and experimental conditions was carried out using the FindMarkers function. The log2 fold changes obtained from the DEG were mapped onto KEGG pathways to visualize which pathways were associated with the differentially expressed genes.

**3. Statistical analysis**

For differential gene expression analysis, the log-fold change threshold was set to 0.25, and *p*-values were adjusted using the Bonferroni correction to control for multiple testing. A minimum expression percentage (min.pct) of 0.1 was applied to identify significant differentially expressed genes in each cluster.
