# Supplementary material for: Spatial Transcriptome Analysis of B7-H4 in Head and Neck Squamous Cell Carcinoma: A Novel Therapeutic Target for Anti-Immune Checkpoint Inhibitors
Source: Head Neck Pathol. 2025 Jun 30;19(1):78. doi: 10.1007/s12105-025-01815-w (PMC12209170; doi:10.1007/s12105-025-01815-w)
Supplement: Supplementary file 5 — Supplementary Material 5: Online Resource 5. Protein and mRNA expression data obtained via spatial analysis from PD-L1 positive cases (S1-6) and B7-H4 positive cases (S4) [file 12105_2025_1815_MOESM5_ESM.docx]

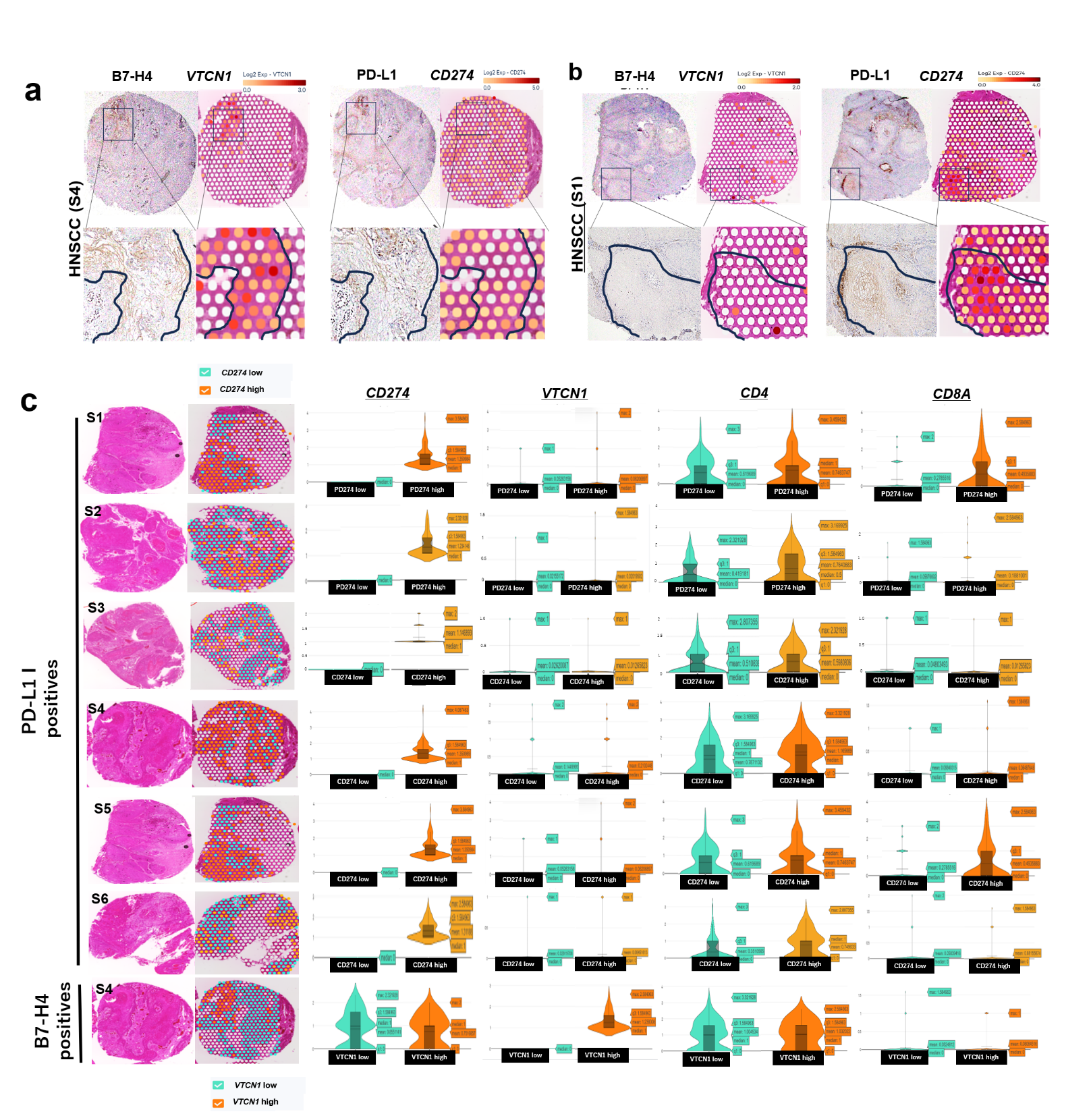
**Online Resource 5.** Protein and mRNA expression data obtained via spatial analysis from PD-L1 positive cases (S1-6) and B7-H4 positive cases (S4)

**(a, b)** Matched distributions of the immunohistochemical expression areas and mRNA upregulated areas for B7-H4 (*VTCN1*) and PD-L1 (*CD274*)**. (c)** mRNA expression according to the spatial analysis in PD-L1 positive cases (S1-6) and B7-H4 positive cases (S4).

S, sample; HNSCC, head and neck squamous cell carcinoma; SIN, squamous intraepithelial neoplasm; NOM, normal mucosa; PD-L1, programmed cell death protein-ligand 1
