# Supplementary material for: Spatial Transcriptome Analysis of B7-H4 in Head and Neck Squamous Cell Carcinoma: A Novel Therapeutic Target for Anti-Immune Checkpoint Inhibitors
Source: Head Neck Pathol. 2025 Jun 30;19(1):78. doi: 10.1007/s12105-025-01815-w (PMC12209170; doi:10.1007/s12105-025-01815-w)
Supplement: Supplementary file 6 — Supplementary Material 6: Online Resource 6. Gene ontology (GO) analysis summary [file 12105_2025_1815_MOESM6_ESM.docx]

**Online Resource 6.** Summary of gene ontology (GO) analysis results

**1. GO: Molecular Functions of18 input genes / 183 annotations before applied cutoff / 19912 genes in category**

**Show 45 more**

|  | ID | Name | Source | pValue | FDR B&H | FDR B&Y | Bonferroni | Genes from Input | Genes in Annotation |
| --- | --- | --- | --- | --- | --- | --- | --- | --- | --- |
| 1 | GO:0004866 | endopeptidase inhibitor activity |  | 5.108E-4 | 1.103E-2 | 6.385E-2 | 9.347E-2 | [3](https://toppgene.cchmc.org/showQueryTerms.jsp?userdata_id=100508e3-563a-44a0-80b1-9ac81fd4cb8b&feature=gof&row=0) | [177](https://toppgene.cchmc.org/showTermDetail.jsp?userdata_id=100508e3-563a-44a0-80b1-9ac81fd4cb8b&category=GeneOntologyMolecularFunction&id=GO:0004866&namespace=) |
| 2 | GO:0140313 | molecular sequestering activity |  | 5.607E-4 | 1.103E-2 | 6.385E-2 | 1.026E-1 | [2](https://toppgene.cchmc.org/showQueryTerms.jsp?userdata_id=100508e3-563a-44a0-80b1-9ac81fd4cb8b&feature=gof&row=1) | [39](https://toppgene.cchmc.org/showTermDetail.jsp?userdata_id=100508e3-563a-44a0-80b1-9ac81fd4cb8b&category=GeneOntologyMolecularFunction&id=GO:0140313&namespace=) |
| 3 | GO:0030414 | peptidase inhibitor activity |  | 5.995E-4 | 1.103E-2 | 6.385E-2 | 1.097E-1 | [3](https://toppgene.cchmc.org/showQueryTerms.jsp?userdata_id=100508e3-563a-44a0-80b1-9ac81fd4cb8b&feature=gof&row=2) | [187](https://toppgene.cchmc.org/showTermDetail.jsp?userdata_id=100508e3-563a-44a0-80b1-9ac81fd4cb8b&category=GeneOntologyMolecularFunction&id=GO:0030414&namespace=) |
| 4 | GO:0061135 | endopeptidase regulator activity |  | 6.671E-4 | 1.103E-2 | 6.385E-2 | 1.221E-1 | [3](https://toppgene.cchmc.org/showQueryTerms.jsp?userdata_id=100508e3-563a-44a0-80b1-9ac81fd4cb8b&feature=gof&row=3) | [194](https://toppgene.cchmc.org/showTermDetail.jsp?userdata_id=100508e3-563a-44a0-80b1-9ac81fd4cb8b&category=GeneOntologyMolecularFunction&id=GO:0061135&namespace=) |
| 5 | GO:0050568 | protein-glutamine glutaminase activity |  | 9.040E-4 | 1.103E-2 | 6.385E-2 | 1.654E-1 | [1](https://toppgene.cchmc.org/showQueryTerms.jsp?userdata_id=100508e3-563a-44a0-80b1-9ac81fd4cb8b&feature=gof&row=4) | [1](https://toppgene.cchmc.org/showTermDetail.jsp?userdata_id=100508e3-563a-44a0-80b1-9ac81fd4cb8b&category=GeneOntologyMolecularFunction&id=GO:0050568&namespace=) |

**2. GO: Biological Process 19 input genes in category / 908 annotations before applied cutoff / 20649 genes in category**

|  | ID | Name | Source | pValue | FDR B&H | FDR B&Y | Bonferroni | Genes from Input | Genes in Annotation |
| --- | --- | --- | --- | --- | --- | --- | --- | --- | --- |
| 1 | GO:2000425 | regulation of apoptotic cell clearance |  | 6.219E-5 | 4.337E-2 | 3.204E-1 | 5.647E-2 | [2](https://toppgene.cchmc.org/showQueryTerms.jsp?userdata_id=100508e3-563a-44a0-80b1-9ac81fd4cb8b&feature=gop&row=0) | [13](https://toppgene.cchmc.org/showTermDetail.jsp?userdata_id=100508e3-563a-44a0-80b1-9ac81fd4cb8b&category=GeneOntologyBiologicalProcess&id=GO:2000425&namespace=) |
| 2 | GO:0098883 | synapse pruning |  | 9.552E-5 | 4.337E-2 | 3.204E-1 | 8.673E-2 | [2](https://toppgene.cchmc.org/showQueryTerms.jsp?userdata_id=100508e3-563a-44a0-80b1-9ac81fd4cb8b&feature=gop&row=1) | [16](https://toppgene.cchmc.org/showTermDetail.jsp?userdata_id=100508e3-563a-44a0-80b1-9ac81fd4cb8b&category=GeneOntologyBiologicalProcess&id=GO:0098883&namespace=) |
| 3 | GO:0000041 | transition metal ion transport |  | 1.568E-4 | 4.744E-2 | 3.506E-1 | 1.423E-1 | [3](https://toppgene.cchmc.org/showQueryTerms.jsp?userdata_id=100508e3-563a-44a0-80b1-9ac81fd4cb8b&feature=gop&row=2) | [116](https://toppgene.cchmc.org/showTermDetail.jsp?userdata_id=100508e3-563a-44a0-80b1-9ac81fd4cb8b&category=GeneOntologyBiologicalProcess&id=GO:0000041&namespace=) |

**3. GO: Cellular Component 19 input genes in category / 85 annotations before applied cutoff / 20915 genes in category**

|  | ID | Name | Source | pValue | FDR B&H | FDR B&Y | Bonferroni | Genes from Input | Genes in Annotation |
| --- | --- | --- | --- | --- | --- | --- | --- | --- | --- |
| 1 | GO:0034774 | secretory granule lumen |  | 2.002E-4 | 6.005E-3 | 3.018E-2 | 1.701E-2 | [4](https://toppgene.cchmc.org/showQueryTerms.jsp?userdata_id=100508e3-563a-44a0-80b1-9ac81fd4cb8b&feature=goc&row=0) | [332](https://toppgene.cchmc.org/showTermDetail.jsp?userdata_id=100508e3-563a-44a0-80b1-9ac81fd4cb8b&category=GeneOntologyCellularComponent&id=GO:0034774&namespace=) |
| 2 | GO:0060205 | cytoplasmic vesicle lumen |  | 2.072E-4 | 6.005E-3 | 3.018E-2 | 1.761E-2 | [4](https://toppgene.cchmc.org/showQueryTerms.jsp?userdata_id=100508e3-563a-44a0-80b1-9ac81fd4cb8b&feature=goc&row=1) | [335](https://toppgene.cchmc.org/showTermDetail.jsp?userdata_id=100508e3-563a-44a0-80b1-9ac81fd4cb8b&category=GeneOntologyCellularComponent&id=GO:0060205&namespace=) |
| 3 | GO:0031983 | vesicle lumen |  | 2.119E-4 | 6.005E-3 | 3.018E-2 | 1.801E-2 | [4](https://toppgene.cchmc.org/showQueryTerms.jsp?userdata_id=100508e3-563a-44a0-80b1-9ac81fd4cb8b&feature=goc&row=2) | [337](https://toppgene.cchmc.org/showTermDetail.jsp?userdata_id=100508e3-563a-44a0-80b1-9ac81fd4cb8b&category=GeneOntologyCellularComponent&id=GO:0031983&namespace=) |
| 4 | GO:0035580 | specific granule lumen |  | 1.431E-3 | 2.814E-2 | 1.414E-1 | 1.217E-1 | [2](https://toppgene.cchmc.org/showQueryTerms.jsp?userdata_id=100508e3-563a-44a0-80b1-9ac81fd4cb8b&feature=goc&row=3) | [62](https://toppgene.cchmc.org/showTermDetail.jsp?userdata_id=100508e3-563a-44a0-80b1-9ac81fd4cb8b&category=GeneOntologyCellularComponent&id=GO:0035580&namespace=) |
| 5 | GO:0030141 | secretory granule |  | 1.655E-3 | 2.814E-2 | 1.414E-1 | 1.407E-1 | [5](https://toppgene.cchmc.org/showQueryTerms.jsp?userdata_id=100508e3-563a-44a0-80b1-9ac81fd4cb8b&feature=goc&row=4) | [1002](https://toppgene.cchmc.org/showTermDetail.jsp?userdata_id=100508e3-563a-44a0-80b1-9ac81fd4cb8b&category=GeneOntologyCellularComponent&id=GO:0030141&namespace=) |

The *DKK1, CST6, C3, RARRES1 TCN1. LCN2, and TGM2,* relating to cellular homeostasis regulatory mechanisms, such as molecular function (endopeptidase inhibitor, ID GO:0004866), biological process (transition metal ion transition, GO:0000041), and cellular component (secretory granule, GO: 0030141).
